# Gut Microbiota Alterations can predict Hospitalizations in Cirrhosis Independent of Diabetes Mellitus

**DOI:** 10.1038/srep18559

**Published:** 2015-12-22

**Authors:** Jasmohan S. Bajaj, Naga S. Betrapally, Phillip B. Hylemon, Leroy R. Thacker, Kalyani Daita, Dae Joong Kang, Melanie B. White, Ariel B. Unser, Andrew Fagan, Edith A. Gavis, Masoumeh Sikaroodi, Swati Dalmet, Douglas M. Heuman, Patrick M. Gillevet

**Affiliations:** 1 Division of Gastroenterology, Hepatology and Nutritiony, George Mason University, Manassas, Virginia, USA; 2 Microbiome Analysis Center, George Mason University, Manassas, Virginia, USA; 3 Department of Microbiology, Virginia Commonwealth University and McGuire VA Medical Center, Richmond, Virginia, USA; 4 Department of Biostatistics, Virginia Commonwealth University and McGuire VA Medical Center, Richmond, Virginia, USA

## Abstract

Diabetes (DM) is prevalent in cirrhosis and may modulate the risk of hospitalization through gut dysbiosis. We aimed to define the role of gut microbiota on 90-day hospitalizations and of concomitant DM on microbiota. Cirrhotic outpatients with/without DM underwent stool and sigmoid mucosal microbial analysis and were followed for 90 days. Microbial composition was compared between those with/without DM, and those who were hospitalized/not. Regression/ROC analyses for hospitalizations were performed using clinical and microbial features. 278 cirrhotics [39% hepatic encephalopathy (HE), 31%DM] underwent stool while 72 underwent mucosal analyses. Ultimately, 94 were hospitalized and they had higher MELD, proton pump inhibitor (PPI) use and HE without difference in DM. Stool/mucosal microbiota were significantly altered in those who were hospitalized (UNIFRAC p< = 1.0e-02). Specifically, lower stool *Bacteroidaceae,* Clostridiales XIV, *Lachnospiraceae, Ruminococcacae* and higher *Enterococcaceae* and *Enterobacteriaceae* were seen in hospitalized patients. Concomitant DM impacted microbiota UNIFRAC (stool, p = 0.003, mucosa,p = 0.04) with higher stool *Bacteroidaceae* and lower *Ruminococcaeae.* Stool *Bacteroidaceaeae* and Clostridiales XIV predicted 90-day hospitalizations independent of clinical predictors (MELD, HE, PPI). Stool and colonic mucosal microbiome are altered in cirrhotics who get hospitalized with independent prediction using stool *Bacteroidaceae* and Clostridiales XIV. Concomitant DM distinctly impacts gut microbiota without affecting hospitalizations.

Hospitalizations in cirrhosis are associated with susceptibility to nosocomial and second infections and can predict a poor prognosis^1^. Type 2 diabetes mellitus (DM) is frequently found in patients with cirrhosis, especially with non-alcoholic steatohepatitis (NASH) and hepatitis C infection, which could worsen the prognosis^2^,^3^,^4^. These hospitalizations, which are most commonly liver-related, could be due to a systemic pro-inflammatory milieu brought on by gut dysbiosis^5^,^6^,^7^. A recent study has shown that the gut microbiota in non-cirrhotic DM patients is significantly different compared to cirrhotic patients^8^. Given the presence of concomitant DM in a large proportion of cirrhotic patients^3^,^4^, it is relevant to its additive impact on the gut microbiota composition and 90-day hospitalizations in cirrhosis. This is partly because DM in non-cirrhotic settings can profoundly impact the gut microbiota with and without the presence of obesity^9^,^10^.

We hypothesized that gut microbiota changes can independently predict the risk of short-term hospitalizations in cirrhosis and this will be modulated by DM independent of the severity of cirrhosis. This issue is important because currently available prognostic markers are often not reliable in predicting these complications^11^. Therefore, the aim of our study was to (i) evaluate the role of gut microbiota in independently predicting 90-day hospitalizations in cirrhosis and (ii) evaluate the impact of DM on this risk through its impact on the gut microbiota.

## Results

### Patient and Outcomes information

#### Demographic information

We considered 335 patients with cirrhosis for this study; 18 had recently consumed alcohol/illicit drugs, 21 refused to participate and 18 were on absorbable antibiotics and were therefore excluded. Ultimately we included 278 cirrhotic patients. The median age was 57 years (IQR 53-61) and BMI was 29 (IQR 26-33). Seventy five percent were men and most were Caucasian (68%) followed by African-American (30%) and Hispanic (2%). The median MELD was 11 (IQR 7-16) and the majority had HCV (40%) followed by alcohol alone (22%), NASH (17%), both alcohol and HCV (13%) and others (8%). Of the 278 patients, 106 (39%) had prior HE (67 on lactulose alone, 39 on both lactulose and rifaximin). Non-selective beta-blockers were being used by 38% of patients while 48% were on PPI therapy. PPI and HE therapy was prescribed together in 68 patients, 38 were only on HE treatment, 72 on PPI only without HE therapy and 100 on neither treatment. 87 cirrhotic patients were diagnosed with DM. Of these 40 were on insulin while the rest were controlled with oral medications. The median duration of DM was 11 years (IQR 7-27) and median HgbA1c was 6.6 (IQR: 5.7-8.1) within the last 6 months. Seventy-two cirrhotics underwent flexible sigmoidoscopy and colonic biopsies. These included 21 subjects with DM (6 on insulin) and 26 with HE (20 controlled on lactulose and 6 on lactulose+rifaximin).

#### Hospitalizations

Of the 278 subjects, 19 were lost to follow-up and 3 had elective hospitalizations. A total of 94 (37%) were non-electively hospitalized within 90 days (median 35, IQR 21-78 days). The major (n = 87) reasons for hospitalization were liver-related (HE = 46, Infection = 14, renal or metabolic reasons = 13, GI bleeding = 10, others = 4). A separate sub-analysis of subjects admitted for HE compared to others was performed since this was the highest sub-group. Those who were hospitalized had a worse cirrhosis severity, were younger, and had a higher PPI use (Table 1). Specifically patients with prior HE and those on rifaximin for their HE had a higher likelihood of admission. An alcoholic etiology was associated with increased hospitalization while the opposite impact was seen with NASH cirrhosis. No overall impact of DM on hospitalizations was observed. On dietary analysis, all patients were non-vegetarians and had statistically equivalent daily caloric intake.

#### Interactions between DM, demographics and cirrhosis

Cirrhotic patients with DM had a higher BMI and proportion with NASH and a lower percent with alcoholic etiology compared to those without DM (Table 2). There was no significant difference in the distribution of HE and MELD score between the groups. In the insulin sub-group when compared to those not using insulin, there was a significantly higher MELD score (15 ± 7.2 vs. 12.0 vs. 5.7, p = 0.03) and had a higher proportion of prior HE (62% vs 35%, p = 0.007).

While DM alone was not associated with an increased rate of hospitalizations, those subjects with advanced or uncontrolled DM who required insulin were at greater risk for hospitalization at 90 days (Table 3).

## Microbiota Results

### Analysis of 90-day hospitalizations and microbiota

**Figure 1:**
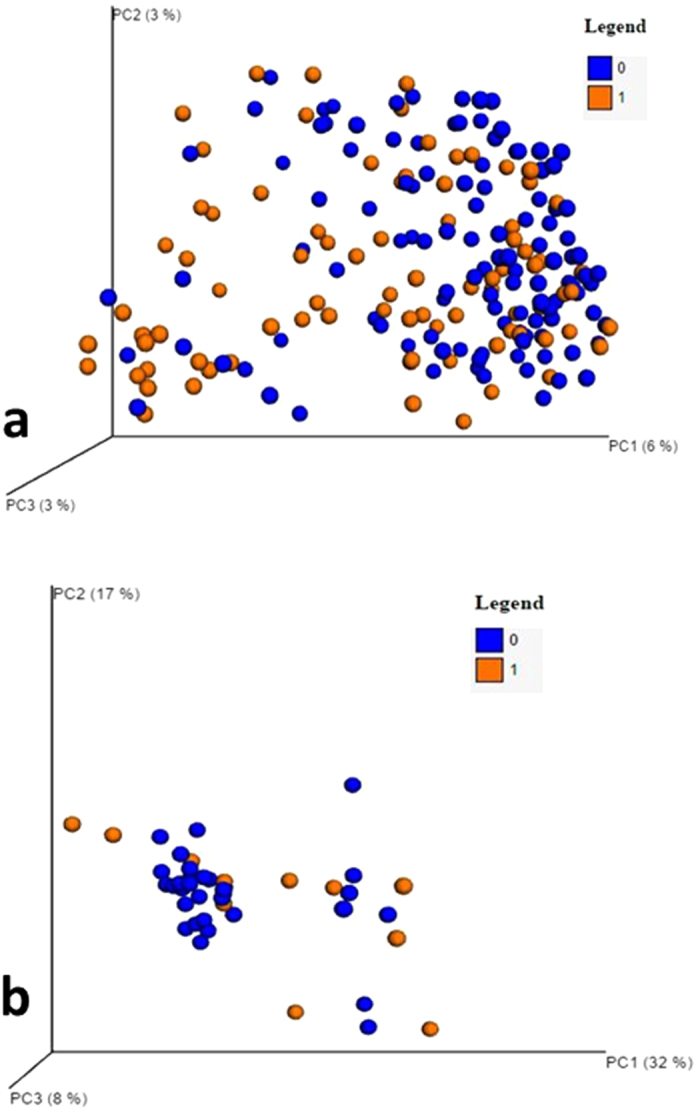
Microbiota differences between groups who were hospitalized or not. (**A,B**) Principal component analysis (PCO) showed a relative clustering of cirrhotics who were not hospitalized (blue dots, coded 0) compared to others (orange dots, coded 1). (**A**) Stool PCO plots with/without hospitalization. (**B**) Sigmoid mucosa PCO plots with/without hospitalization.

The overall UNIFRAC analysis showed significant differences in stool microbiota between those hospitalized compared to others (p <=1.0e-02). There was a relative clustering of those not hospitalized compared to those hospitalized on PCA (Fig. 1A). On metastats and Kruskal-Wallis tests specific microbial families were significantly different in cirrhotics who developed a 90-day hospitalization compared to the rest (Table 3, Figure S1).

UNIFRAC also demonstrated a significant difference in mucosal microbial distribution between cirrhotics were hospitalized compared to others (p = 0.02). There was also a relative clustering of those who were not hospitalized on PCA (Fig. 1B). When individual families were evaluated, only a lower *Porphyromonadaceae*, and a higher relative abundance of two families in *Proteobacteria*, *Succinovibrionaceae* and *Cystobacteriaeae* were associated with admission using Metastats and LeFSe (Figure S4). PPI use was more prevalent in cirrhotics with more advanced liver disease, and was associated with dysbiosis that reflected severity. PPI users in addition had a higher *Streptococcaceae* relative abundance^12^ (Table S1, Figures S3 and S6), which persisted even when PPI use was studied in the context of HE therapy (Table S3). PPI use with HE therapy was also associated with a higher *Bacteroidaceae* and lower *Enterococcaceae* relative abundance.

Patients who were admitted for HE were not significantly different from those admitted for reasons other than HE on cirrhosis severity, demographics, and microbial composition apart from an increase in *Veillonellaceae* and a higher proportion with prior HE and PPI use (Table S2). There was a non-significant trend towards lower dysbiosis in subjects who were ultimately hospitalized for HE compared to others.

### Impact of DM on microbiota

Bonferroni-corrected diabetes-weighted UNIFRAC values showed a significant difference in the stool microbiota (p = 0.003) and mucosal microbiota (p = 0.04) families. When insulin-using cirrhotics with DM were compared to those with DM but not using insulin on diabetes-weighted UNIFRAC, there was a significant difference in mucosal microbiota (p = 0.006) but not in the stool microbiota distribution (p = 0.56).

### Stool microbiota

There was no significant differences at the phylum level in stool microbiota median relative abundances between patients with/without DM (Bacteroidetes 46 vs 41%, Firmicutes 35 vs 41%, Proteobacteria 1.5 vs 1.2%, Firmicutes/Bacteroidetes ratio: 0.74 vs 0.76, all p > 0.05). At the family level there was a higher *Bacteroidaceae* and *Streptococcaceae* and lower relative abundance of *Ruminococcaeae* and *Veillonellaceae* (Table 3, Figure S2). The autochthonous bacteria abundance (*Ruminococcae*+Clostridiales XIV+*Lachnospiraceae* 9% vs. 26%, p = 0.005) was further reduced in insulin-using patients but no other changes were seen.

### Sigmoid mucosa

No significant mucosal phylum relative abundance changes were seen between group and a pattern of mucosal families distinct from stool changes were seen in DM (Table 2, Figure S5). In insulin-using cirrhotics there was an increased *Enterococcaceae* relative abundance (3% vs. 0%, p = 0.01).

### Logistic regression predicting 90-day hospitalizations

On binary logistic regression, 90-day hospitalizations were predicted by *Bacteroidaceaeae* (OR: 0.25, CI: 0.07–0.89, p = 0.02) and Clostridiales XIV (OR: 0.03, CI: 0.01–0.99, p = 0.04) relative abundance independent of MELD (OR:1.16, 1.08–1.25, p < 0.001), prior HE (OR:2.7, CI: 1.3–5.6, p = 0.01) and PPI use (OR:2.2, CI:1.1–4.5, p = 0.2). Etiology, age, DM and other microbes were not predictive. A further analysis was performed using ROC analysis using this above regression formula with and without the microbiota. Using the base model consisting of MELD score, prior HE and PPI use, the AUC was 0.80 (0.74–0.87 95% confidence interval). When the significant microbial families, *Bacteroidaceaeae* and Clostridiales XIV were added to this ROC analysis, the AUC significantly increased to 0.83 (0.76–0.89, p = 0.05).

## Discussion

We found that cirrhotic subjects who required non-elective 90-day hospitalization had a different microbial profile that could add to the current models for this prediction. We also found that although DM in the presence of cirrhosis alters the mucosal and stool microbiota compared to cirrhotics without DM, it does not add to the 90-day hospitalization risk.

Hospital admissions are a growing healthcare burden in cirrhosis that requires urgent attention^5^,^11^,^13^. Clinical models of these admissions center on cirrhosis severity and complications, which may require refinement using further patho-physiological tools^11^. In our study, we found that gut microbiota alterations can independently add to this predictive capacity beyond cirrhosis severity and medication usage. We found significant differences in the stool and sigmoid mucosal microbiota composition at the family level between those with and without hospitalizations. Replicating prior studies, we found a significantly lower autochthonous bacterial relative abundance and increased potentially pathogenic microbiota in advanced cirrhotics, who in turn were more likely to be hospitalized^14^,^15^. An independent contribution of a reduced relative abundance of *Bacteroidaceae* and Clostridiales XIV towards this outcome was found. These results extend prior studies that have evaluated hospitalized cirrhotic patients for either 30-day mortality and organ failure or those with established acute-on-chronic liver failure with early mortality, into the outpatient cirrhosis realm^14^,^16^. These studies showed that different bacterial families, *Lachnospiraceae*, Clostridiales XIV, *Ruminococcaceae* and *Pasteurellaceae* were associated with short-term mortality compared to *Bacteroidaceae* and Clostridiales cluster XIV in the current study evaluating relatively longer-term events. Similar results were also found with saliva-related microbiota in the prediction of hospitalizations in a smaller set of patients^17^. However the greater sample size and quantum higher bacterial concentration in the stool compared to saliva would potentially make this a more robust observation.

Different bacterial functions and roles may be relevant in these differences over the short and long-term prognoses. Clostridiales XIV, *Lachnospiraceae* and *Ruminococcaceae* relative abundance has been shown to parallel liver disease severity and they have been potentially beneficial impacts on bile acids and short-chain fatty acids, which could reduce colonic pH and support the intestinal barrier^18^,^19^. The role of *Bacteroidaceae* may be more nuanced. *Bacteroidaceae* are a large family within Bacteroidetes phylum which usually form 20–30% of bacterial abundance in cirrhotic subjects^8^,^14^. Cirrhotics who were ultimately hospitalized had a significant reduction in only two families in Bacteroidetes, *Bacteroidaceae* and *Porphyromonadaceae* but not others such as *Prevotellaceae* or *Rikenellaceae*. This indicates that this is not simply a reduction in the whole phylum but specific families, especially since the Firmicutes/Bacteroidetes ratio was not significantly different between groups. Members of *Bacteroidaceae* are relatively resistant to antibiotics, produce a weak endotoxin and can protect commensal bacteria against antibiotics^20^. Indeed an environment low in *Bacteroidetes* has been shown to promote the growth of *C.difficile*^21^. Therefore this reduction in *Bacteroidaceae* may indicate a gut milieu prone towards development of further insults regardless of HE and MELD score. The relative increase in relative abundance of *Bacteroidaceae* in NASH and DM cirrhotics compared to alcoholics and other etiologies of cirrhosis could also explain the historically higher rate of infections in alcoholic cirrhotic subjects compared to NASH patients^14^,^22^.

The relationship between sigmoid mucosal *Porphyromonadaceae* reduction, increase in families belonging to *Proteobacteria* and subsequent hospitalizations is novel in this study. *Porphyromonadaceae* are usually of oral origin that have been associated with higher inflammation, progression of fatty liver disease and cognitive dysfunction in human and animal studies^23^,^24^. Members of *Proteobacteria* are usually increased in the stool of cirrhotic subjects and are linked with endotoxemia, but our study extended this onto the mucosa and linked them with clinically-relevant outcomes^15^. A recent study showed that in cirrhotics that have already been hospitalized ultimately achieve a microbial pattern i.e. significantly lower *Bacteroidaceae, Porphyromonadaceae* and Clostridiales XIV relative abundance compared to outpatients, indicating the complicity of these changes in promoting future adverse outcomes^14^.

It is also interesting that despite being on medications that improve overall outcomes by altering gut microbiota composition and function i.e. lactulose and rifaximin, patients were still prone to development of hospitalizations that were predicted by microbial changes^25^,^26^,^27^. A recent study has found that the probiotic VSL#3 reduced overall hospitalizations but not specifically HE episodes, compared to placebo in patients who had recovered from HE but were not on lactulose^28^. This randomized trial clearly sets the standard for beneficial microbial manipulation but did not study the probiotics in the context of lactulose, the standard of care, and did not evaluate the microbiome. However, our underlying microbial differences between those who were hospitalized or not but not within those who were hospitalized for HE compared to other conditions, could partly explain their results^7^. Therefore the impact of the microbiota (decreased Clostridiales XIV and *Bacteroidaceae*) may prime the milieu for future insults that are result in admission regardless of the proximate cause. Future research into therapies that can beneficially alter the microbiota and prevent these outcomes in cirrhotics already taking standard of care treatment (lactulose and rifaximin) is required.

In cirrhotics with concomitant DM compared to those without it, we found a significantly different microbial composition at the stool and mucosal level. Specifically families in stool showed an increased relative abundance of *Bacteroidaceae*, *Veillonellaceae, Streptococcaceae* and *Eubacteriaceae* with a decrease in autochthonous *Ruminococcaceae*. This pattern has been shown in prior NASH cirrhosis experience, which was over-represented in this population, as well as in non-cirrhotic DM studies and studies of obesity^10^,^14^,^29^,^30^. The modulation of the microbiome with NASH, DM and obesity, can now be interpreted in the context of concomitant cirrhosis. Interestingly, this pattern is different from advancing cirrhosis and those who ultimately required hospitalization; it is likely a DM-related change in microbial composition^31^. However despite an altered microbiota composition in the sigmoid mucosa and the stool, DM in itself did not predispose to higher 90-day hospitalizations. However, the subgroup on insulin was indeed associated with a higher hospitalization rate, which could possibly due to a worse DM control and accompanying dysbiosis. The lack of effect on hospitalization overall in all DM patients may be due to relatively shorter follow-up compared to prior studies that did show an impact of DM on prognosis^2^. We limited our follow-up to 90 days to minimize variability within the microbiota from the baseline and because that is the validity of the MELD score^32^.

Replicating prior studies, we found that PPI use was a significant predictor of admissions and were more likely used in those with more advanced liver disease^33^,^34^. In addition to the generalized dysbiosis, there was a significant increase in *Streptococcacae* relative abundance, presumably of a salivary origin, with PPI use as prior studies have also shown^12^,^35^. This specific increase in *Streptococcacae* in PPI-using subjects also highlights the exceedingly complex gut milieu that is influenced differently by each medication. Interestingly this trend persisted even in the presence of HE therapy. However, despite controlling for all other important variables, PPI use remained significantly predictive of admissions.

Although this is the largest experience of mucosal microbiota to date in cirrhosis, changes in mucosal microbiota were not as predictive as stool for hospitalizations. As expected families from *Proteobacteria* had a higher relative abundance in the mucosa of those who were hospitalized, that demonstrates a different pattern of dysbiosis from that seen in the stool. This could be due to a relative stability of mucosal microbiota compared to changes in stool over time with factors such as diet or could be due to the relatively smaller sample of patients who underwent sigmoid biopsies. However from a practical standpoint, the relative non-invasiveness of stool collection compared to sigmoid mucosa, is encouraging towards using these samples, rather than the mucosal ones, for prediction of hospitalizations.

The study is a descriptive and cross-sectional analysis of microbiota to predict outcomes over 90 days, which did not study variations over time. However, in a prior study we found that gut microbiota track the underlying disease process and are stable over time^14^. There are also several other factors, including genetic variations and changes in microbial functionality, that could also impact the development of further complications in cirrhosis, that were not specifically assessed^36^. While the changes in bacterial subgroups are not as striking as those found in studies comparing cirrhotics with non-cirrhotic groups or with healthy controls^8^,^14^,^15^, it is important to realize that these were found in the context of our population of only cirrhotic subjects and were independently related to poor clinical outcomes despite controlling for available biomarkers. The use of MTPS also limited us to a relative smaller depth compared to metagenomic sequencing^37^; future studies are needed to evaluate these for long-term clinical outcomes. Despite these limitations, we were able to define a distinct microbial pattern in concomitant DM and in cirrhotics who were ultimately hospitalized.

The results demonstrate that gut and mucosal microbiota are altered in cirrhotic subjects who are non-electively hospitalized within 90 days regardless of the cause of hospitalization. This pattern is different from that induced by concomitant DM. Stool microbiota changes can enhance the predictive capability of current traditional biomarkers in the prediction of 90-day hospitalizations. Further studies into beneficial microbial modulation in cirrhotic patients to prevent hospitalizations are needed on the background of standard of care treatments.

## Methods

We prospectively enrolled consecutive outpatients with cirrhosis (diagnosed through liver biopsy, or through the presence of varices or signs of portal hypertension in the setting of chronic liver disease, or with features of frank decompensation) after informed consent. We excluded cirrhotic patients with type 1 diabetes, HIV infection, those on absorbable antibiotics and those who were actively drinking, receiving absorbable antibiotics or probiotics (within the last 3 months). Patients with cirrhosis included those with concomitant DM in whom the medications (insulin or non-insulin medications) and duration of diabetes were recorded. Data collected was related to cirrhosis severity (MELD, model for end-stage liver disease, a logarithmic validated cirrhosis severity score)^32^, prior history of hepatic encephalopathy (HE), use of concomitant medications including beta-blockers, proton pump inhibitors (PPI), rifaximin and lactulose (both used for HE therapy). At enrollment a detailed 3-day dietary history through recall was performed and average daily calories were noted. Subjects were then systematically followed for 90 days after enrollment for non-elective hospitalizations. Hospitalizations were identified using active follow-up, chart review and phone calls in case subjects were not seen within 90 days. These episodes were classified as liver-related (HE, infections, ascites/fluid-electrolyte, gastrointestinal bleeding or others) or unrelated hospitalizations.

### Sample collection and processing

All subjects gave a fresh stool sample which was collected using a Parapak stool collection kit with 5 ml of RNALater, from which DNA was extracted within 24 hours of collection. A subset of patients who had given stool also underwent an un-prepped sigmoidoscopy with biopsy of the sigmoid colon. DNA was extracted for 16SRNA pyrosequencing from the stool and sigmoid biopsies. The stool and sigmoid mucosal microbiota were characterized using standard techniques.

### Microbiota

DNA extracted from stool and sigmoid colonic mucosa using published techniques^38^. We first used Length Heterogeneity PCR (LH-PCR) fingerprinting of the 16S rRNA to rapidly survey our samples and standardize the community amplification. We then interrogated the microbial taxa associated using Multitag Pyrosequencing (MTPS) 39. This technique allows the rapid sequencing of multiple samples at one time.

### Microbiome Community Fingerprinting

About 10 ng of extracted DNA was amplified by PCR using a fluorescently labeled forward primer 27F (5′-(6FAM) AGAGTTTGATCCTGGCTCA G-3′) and unlabeled reverse primer 355R’ (5′-GCTGCCTCCCGTAGGAGT-3′). Both primers are universal primers for bacteria. The LH-PCR products were diluted according to their intensity on agarose gel electrophoresis and mixed with ILS-600 size standards (Promega) and HiDi Formamide (Applied Biosystems, Foster City, CA). The diluted samples were then separated on a ABI 3130xl fluorescent capillary sequencer (Applied Biosystems, Foster City, CA) and processed using the Genemapper™ software package (Applied Biosystems, Foster City, CA). Normalized peak areas were calculated using a custom PERL script and operational taxonomic units (OTUs) constituting less than 1% of the total community from each sample were eliminated from the analysis to remove the variable low abundance components within the communities.

### MTPS^39^

Specifically, we have generated a set of 96 emulsion PCR fusion primers that contain the 454 emulsion PCR linkers on the 27F and 355R primers and a different 8 base “barcode” between the A adapter and 27F primer. Thus, each fecal sample was amplified with unique bar-coded forward 16S rRNA primers and then up to 96 samples were pooled and subjected to emulsion PCR and pyrosequenced using a GS-FLX pyrosequencer (Roche). Data from each pooled sample were “deconvoluted” by sorting the sequences into bins based on the barcodes using custom PERL scripts. Thus, we were able to normalize each sample by the total number of reads from each barcode. We have noted that ligating tagged primers to PCR amplicons distorts the abundances of the communities and thus it is critical to incorporate the tags during the original amplification step.

### Microbiome Community Analysis

We identified the taxa present in each sample using the Bayesian analysis tool in Version 10 of the Ribosomal Database Project (RDP10). The abundances of the bacterial identifications were then normalized using a custom PERL script and genera present at >1% of the community were tabulated. We chose this cutoff because of our *a priori* assumption that genera present in < 1% of the community vary between individuals and have minimal contribution to the functionality of that community and 2,000 reads per sample will only reliably identify community components that are greater than 1% in abundance.

### Statistical analysis

The demographics, DM status, MELD score, HE status and concomitant medications were compared between groups. The overall changes in microbial abundance between patients who experienced a non-elective hospitalization within 90 days were compared to other subjects. In addition, microbiota differences between subjects with and without DM were compared. On an entire microbiota level, this was performed using UNIFRAC with principal component analyses for stool and mucosal microbiome^40^. Version 1.3.0 of Quantitative Insights into Microbial Ecology (QIIME) was used with weighted p-values according to hospitalization and then for DM. To identify which specific bacterial families were associated with these outcomes, we analyzed the microbiota of the stool and sigmoid mucosa using Metastats, Linear Discriminant Analysis (LFSe) and Kruskal-Wallis^41^. LFSe is a stringent evaluation of differences that only takes into account differences >2 standard deviations between groups. A false discovery rate of q<0.05 was used for all analyses.

We also analyzed microbiota differences between cirrhotic patients on insulin compared to DM cirrhotics who were not on insulin, between those on and not on PPI and those who were hospitalized for HE compared to those hospitalized for other reasons.

Logistic regression with 90-day hospitalizations as the outcome was performed using variables that were significant with p < 0.1 on univariate regression. Variables tested with prior HE, MELD score, DM, alcoholic etiology, PPI use, rifaximin use, beta-blocker use, individual bacterial family relative abundances. The regression equation with clinical and bacterial families predicting 90-day hospitalization was used to plot an ROC curve. The area-under the curve (AUC) was compared with the regression equation with only clinical parameters to evaluate the specific contribution of the microbiota to the 90-day hospitalization risk.

The protocol was approved by the Institutional Review Boards at the VCU Medical Center and the Richmond VA Medical Center and the methods were carried out in accordance with the approved guidelines.

## Additional Information

**How to cite this article**: Bajaj, J. S. *et al.* Gut Microbiota Alterations can predict Hospitalizations in Cirrhosis Independent of Diabetes Mellitus. *Sci. Rep.* **5**, 18559; doi: 10.1038/srep18559 (2015).

## Supplementary Material

Supplementary Information

## Figures and Tables

**Table 1:** Comparison between subjects hospitalized and not hospitalized within 90 days.

*p < 0.05, **p < 0.01, ***p < 0.001	Hospitalized within 90 days (n = 94)	Not hospitalized within 90 days (n = 162)
Age	56 ± 8*	58 ± 5
BMI	29 ± 6	31 ± 5
Daily calories	2947 ± 659	2902 ± 803
Alcoholic etiology	31%*	22%
NASH etiology	11%*	21%
MELD score	17 ± 8***	10 ± 4
With prior HE	64%***	23%
Additionally on rifaximin	33%***	21%
Type 2 Diabetes	33%	30%
Diabetes on insulin (within DM group)	51%*	28%
Non-selective beta-blockers	51%	41%
PPI therapy	68%***	39%

**Table 2:** Comparison between cirrhotic subjects with and without type 2 diabetes.

*p < 0.05, ***p < 0.001	Without DM (n = 191)	DM (n = 87)
Age	56.0 ± 5.9	56.1 ± 5.7
BMI	28.9 ± 5.8	32.1 ± 5.8*
Daily calories	2866 ± 792	3012 ± 952
Alcoholic etiology	43%	19%***
NASH etiology	6%	40%*
MELD score	12.5 ± 6.4	12.7 ± 6.4
Prior HE	37%	42%
Additionally on rifaximin	18%	23%
Non-selective beta-blockers	42%	45%
PPI use	50%	49%
Stool Families		
*Bacteroidetes_Bacteroidaceae*	34.2*	27.3
*Firmicutes_Eubacteriaceae*	1.2*	0.0
*Firmicutes_Ruminococcaceae*	5.5*	9.2
*Firmicutes_Veillonellaceae*	1.4*	0.0
*Firmicutes_Streptococcaceae*	2.0*	0.0
Cirrhosis dysbiosis ratio	0.83	0.77
Mucosal Families		
*Actinobacteria_Streptomycetae*	2.0*	0.0
*Firmicutes_Clostridiacaeae*	2.0*	0.0
*Bacteroidetes_Prevotellaceae*	1.1*	6.1
*Fusobacteria_Fusobacteriaceae*	0.0*	2.0

Significantly different microbial taxa are presented as % median relative abundance.

**Table 3:** Specific significant stool microbiota between cirrhotic subjects with and without 90-day Hospitalization: *p < 0.05, **p < 0.01, ***p < 0.001.

% median stool relative abundance	Hospitalized within 90 days (n = 94)	Not hospitalized within 90 days (n = 162)
Phylum		
*Bacteroidetes*	32.1*	46.1
*Firmicutes*	43.9	38.2
*Proteobacteria*	1.0	0.0
*Actinobacteria*	1.0	1.0
*Fusobacteria*	0.0	0.0
*Firmicutes/Bacteroidetes* ratio	0.75	0.74
Phylum_Family		
*Bacteroidetes_Bacteroidaceae*	12.2*	26.0
*Bacteroidetes_Porphyromonadaceae*	0.0*	2.6
*Bacteroidetes_Prevotellaceae*	6.1	5.1
*Bacteroidetes_Rikenelleaceae*	1.8	2.6
*Firmicutes_Lactobacillaceae*	8.4*	3.9
*Firmicutes_Enterococcaceae*	8.9*	1.7
*Firmicutes_Clostridiales XIV*	0.0*	3.2
*Firmicutes_Lachnospiraceae*	7.8*	14.8
*Firmicutes_Ruminococcaceae*	3.4*	7.0
*Firmicutes_Veillonellaceae*	1.6	1.4
*Proteobacteria_Enterobacteriaceae*	7.8*	3.4
*Proteobacteria_Pasteurellaceae*	0.7*	0.3
Cirrhosis Dysbiosis ratio	0.59*	0.81
