# Erratum: Gut Microbiota Alterations can predict Hospitalizations in Cirrhosis Independent of Diabetes Mellitus

**DOI:** 10.1038/srep20447

**Published:** 2016-01-29

**Authors:** Jasmohan S. Bajaj, Naga S. Betrapally, Phillip B. Hylemon, Leroy R. Thacker, Kalyani Daita, Dae Joong Kang, Melanie B. White, Ariel B. Unser, Andrew Fagan, Edith A. Gavis, Masoumeh Sikaroodi, Swati Dalmet, Douglas M. Heuman, Patrick M. Gillevet

Scientific Reports 5: Article number: 1855910.1038/srep18559; published online: 12222015; updated: 01292016

In the original version of this Article, there were errors in Affiliation 1 which was incorrectly listed as ‘Division of Gastroenterology, Hepatology and Nutritiony, George Mason University, Manassas, Virginia, USA’. The correct affiliation is listed below:

Division of Gastroenterology, Hepatology and Nutrition, Virginia Commonwealth University and McGuire VA Hospital, Richmond, Virginia, USA

These errors have now been corrected in the PDF and HTML versions of the Article.

