# Supplementary material for: Gut Microbiota Alterations can predict Hospitalizations in Cirrhosis Independent of Diabetes Mellitus
Source: Sci Rep. 2015 Dec 22;5:18559. doi: 10.1038/srep18559 (PMC4686976; doi:10.1038/srep18559)
Supplement: Supplementary Information [file srep18559-s1.pdf]

Gut Microbiota Alterations can predict Hospitalizations in Cirrhosis Independent of Diabetes Mellitus

Jasmohan S Bajaj*[1], Naga S Betrapally[2], Phillip B Hylemon[3], Leroy R Thacker[4], Kalyani Daita[1], Dae Joong Kang[1], Melanie B White[1], Ariel B Unser[1], Andrew Fagan[1], Edith A Gavis[1], Masoumeh Sikaroodi[2], Swati Dalmet[2], Douglas M Heuman[1], Patrick M Gillevet[2]

[1]Division of Gastroenterology, Hepatology and Nutrition, [3]Department of Microbiology, [4]Department of Biostatistics, Virginia Commonwealth University and McGuire VA Medical Center, Richmond, Virginia, USA

[2]Microbiome Analysis Center, George Mason University, Manassas, Virginia, USA

Supplementary Data:

Table S1: Comparison of subjects on and not on Proton Pump Inhibitor (PPI) therapy

*p<0.05,**p<0.01, ***p<0.001	Not on PPI (n=138)	PPI (n=140)
Age	56.7±6.8	56.2±6.4
BMI	29.6±6.3	29.9±5.7
Alcoholic etiology	20%	23%
NASH etiology	19%	14%
MELD score	11.3±5.3	13.9±7.1**
Hospitalized within 90 days	28%	52%***
Prior HE	25%	49%***
Additionally on rifaximin	11%	25%**
Non-selective beta-blockers	28%	48%***
Diabetes	30%	31%
Stool phylum		
Bacteroidetes	43.0	34.7*
Firmicutes	38.4	48.8*
Proteobacteria	0.0	1.2
Actinobacteria	1.1	1.1
Fusobacteria	0.0	0.0
Firmicutes/Bacteroidetes ratio	0.79	1.1*
Stool Phylum_Family		
Bacteroidetes_Bacteroidaceae	26%	16%
Bacteroidetes_Porphyromonadaceae	2.4%	1.0%*
Firmicutes_Streptococcaceae	0.0%	2.4%***
Firmicutes_Enterococcaceae	1.0%	6.4%**

Firmicutes_Clostridiales XIV	3.1%	2.3%*
Firmicutes_Lachnospiraceae	15.5%	11.6%**
Firmicutes_Ruminococcaceae	7.9%	4.5%***
Firmicutes_Veillonellaceae	1.4%	13.3%
Proteobacteria_Enterobacteriaceae	4.0%	5.0%
Cirrhosis dysbiosis ratio (low is worse)	1.2	0.8

Table S2: Differences between subjects hospitalized for Hepatic Encephalopathy (HE) compared to others

*p<0.05,**p<0.01, ***p<0.001	Not hospitalized for HE (n=48)	Hospitalized for HE (n=46)
Age	56.6±7.0	55.8±7.5
BMI	31.0±6.8	27.4±5.8*
Alcoholic etiology	18%	33%*
NASH etiology	15%	8%
MELD score	16.4±8.3	15.8±5.8
Prior HE	51%	75%*
Non-selective beta-blockers	50%	50%
PPI use	50%	87%**
Diabetes	30%	31%
Stool phylum		
Bacteroidetes	29.8	39.2
Firmicutes	47.1	40.5
Proteobacteria	1/8	0.0
Actinobacteria	1.1	1.2
Fusobacteria	0.0	0.0
Firmicutes/Bacteroidetes ratio	0.71	0.53
Stool Phylum_Family		
Bacteroidetes_Bacteroidaceae	8.7%	16.2%
Bacteroidetes_Porphyromonadaceae	1.0%	0.0%
Firmicutes_Streptococcaceae	3.1%	2.9%
Firmicutes_Clostridiales XIV	1.0%	1.0%
Firmicutes_Lachnospiraceae	7/2%	9.1%

Firmicutes_Ruminococcaceae	2.6%	4.8%
Firmicutes_Veillonellaceae	1.4%	2.5%*
Proteobacteria_Enterobacteriaceae	10.6%	5.5%
Cirrhosis dysbiosis ratio	0.64	0.61

Table S3: Comparison of cirrhosis details and stool microbiota based on interaction of PPI and HE therapy

*p<0.05,**p<0.01, ***p<0.001	On neither (n=100)	On PPI only (n=72)	On HE therapy only (n=38)	Both PPI and HE therapy (n=68)
Age	58.3±6.8	53.3±6.7	51.5±8.2	55.9±6.0
BMI	30.2±5.9	30.4±5.8	27.1±6.6	30.2±6.0
Alcoholic etiology	20%	23%	31%	18%
NASH etiology	21%	17%	8%	9%
MELD score***	10.6±5.0	12.6±6.8	14.6±4.6	16.1±6.4
Hospitalized within 90 days***	16%	50%	81%	63%
Diabetes	29.6%	38%	31%	23%
Stool phylum				
*Bacteroidetes**	49.4%	38.1%	37.9%	32.0%
*Firmicutes**	32.6%	44.4%	25.8%	51.7%
Proteobacteria	0.0%	1.5%	3.3%	1.3%
Firmicutes/Bacteroidetes ratio	0.58	0.91	0.37	1.3
Stool Phylum_Family				
*Bacteroidetes_Bacteroidaceae**	27%	21%	4%	10%
*Bacteroidetes_Porphyromonadaceae**	2.5%	1.4%	2.0%	0.0%
*Firmicutes_Streptococcaceae****	0.0%	2.0%	0.0%	1.6%
*Firmicutes_Enterococcaceae**	0.0%	5.0%	3.8%	8.9%
*Firmicutes_Clostridiales XIV**	2.7%	2.6%	0.0%	1.7%
*Firmicutes_Lachnospiraceae**	15.1%	12.7%	9.8%	9.3%
*Firmicutes_Ruminococcaceae****	7.1%	3.7%*	4.6%	4.2%

Firmicutes_Veillonellaceae	1.3%	1.4%	1.8%	1.5%
Proteobacteria_Enterobacteriaceae	3.8%	5.0%	13.2%	5.1%
Cirrhosis dysbiosis ratio (low is worse)	1.09	0.75	0.37	0.78

Supplementary Figures

Legends common to all figures: LDA score represents log changes in relative bacterial family representation. The cladogram shows the phylogenetic relationship between the bacterial families, LDA: linear discriminant analysis, PPI: proton pump inhibitor

Figure S1: LEfSe predictions for bacterial families found in stool for patients hospitalized within 90 days. Bars in the green indicate bacterial families represented in hospitalized patients whereas those in red bacterial families represented in patients who were not hospitalized.

Figure S2: LEfSe predictions for bacterial families found in stool for patients with diabetes. Bars in the green indicate bacterial families represented in patients with diabetes whereas those in red bacterial families represented in patients without diabetes.

Figure S3: LEfSe predictions for bacterial families found in stool for patients treated with PPI. Bars in the green indicate bacterial families represented in patients treated with PPI whereas those in red bacterial families represented in patients with no PPI treatment.

Figure S4: LEfSe predictions for bacterial families found in mucosa for patients hospitalized within 90 days. Bars in the red indicate bacterial families represented in hospitalized patients.

Figure S5: LEfSe predictions for bacterial families found in mucosa for patients with diabetes. Bars in the red indicate bacterial families represented in patients with diabetes.

Figure S6: LEfSe predictions for bacterial families found in mucosa for patients treated with PPI. Bars in the red indicate bacterial families represented in patients treated with PPI.

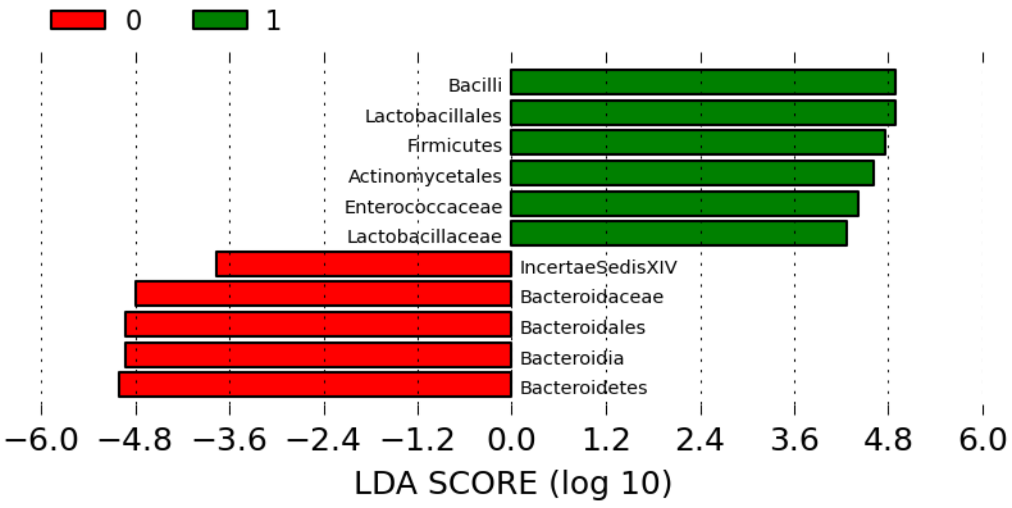

Figure S1

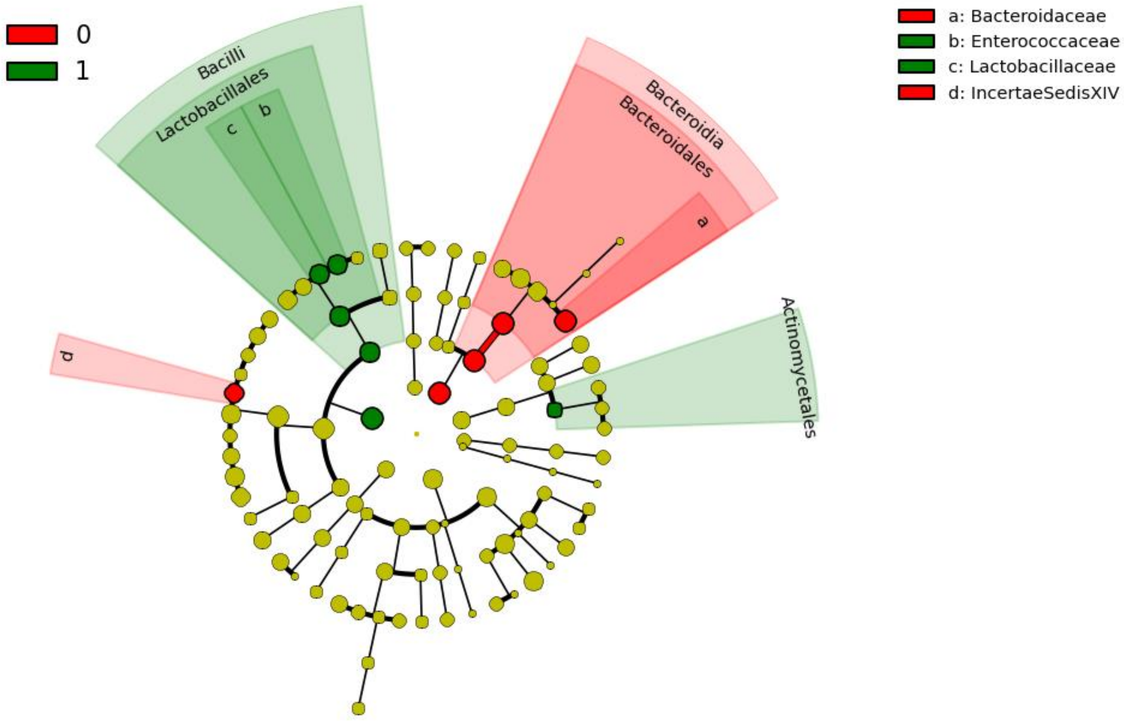

Bacterial Families in stool from patients hospitalized in 90 days

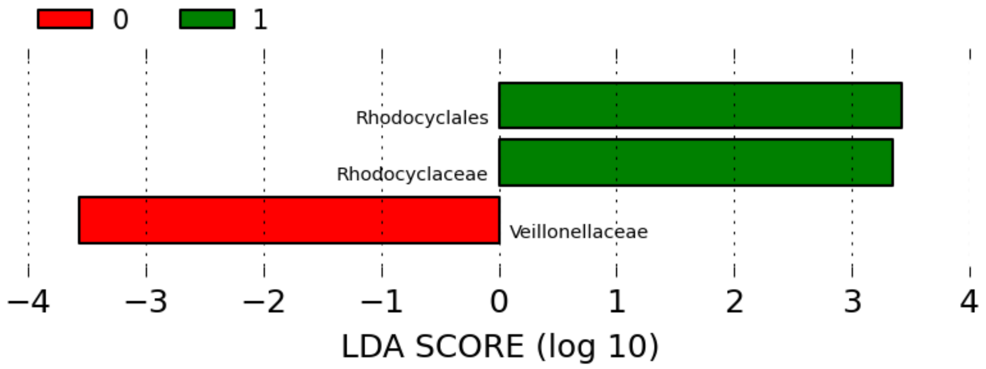

Figure S2

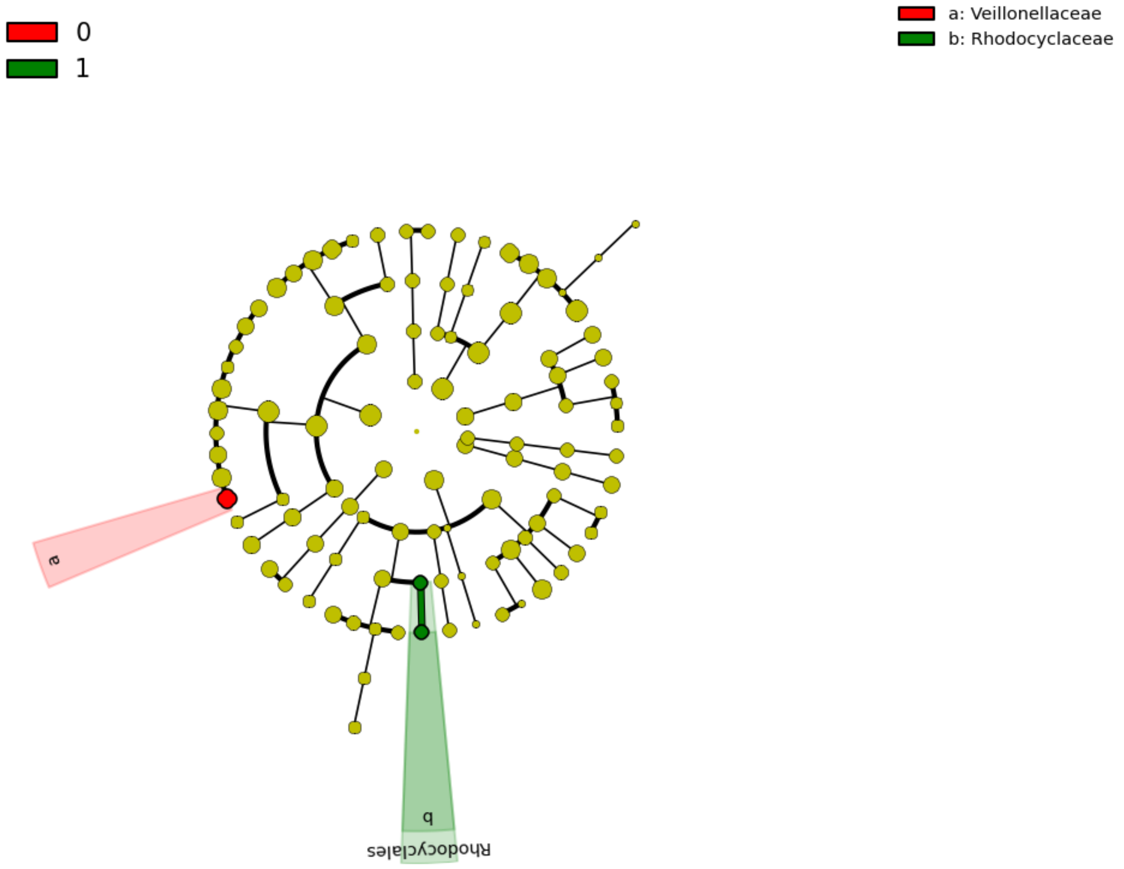

Bacterial Families in stool from patients with diabetes

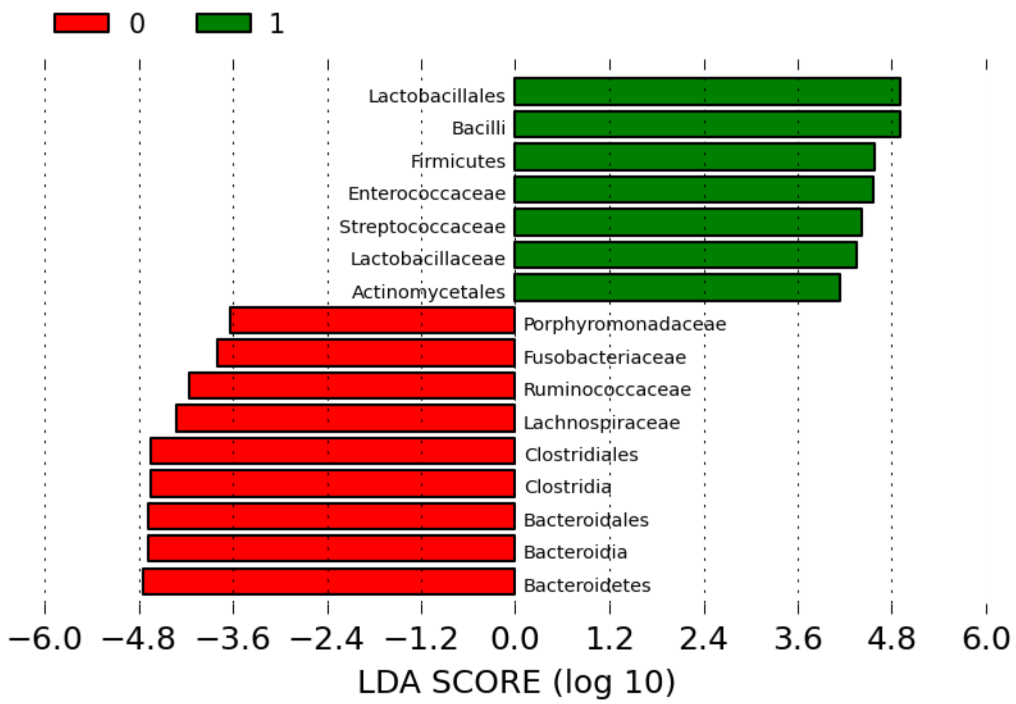

Figure S3

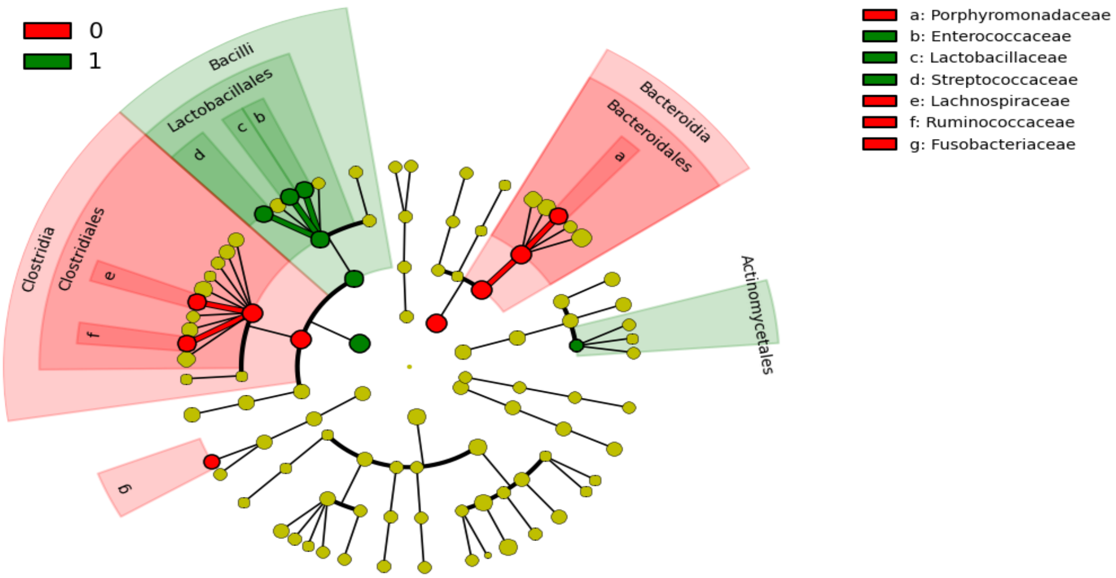

Bacterial Families in stool from patients treated with PPI

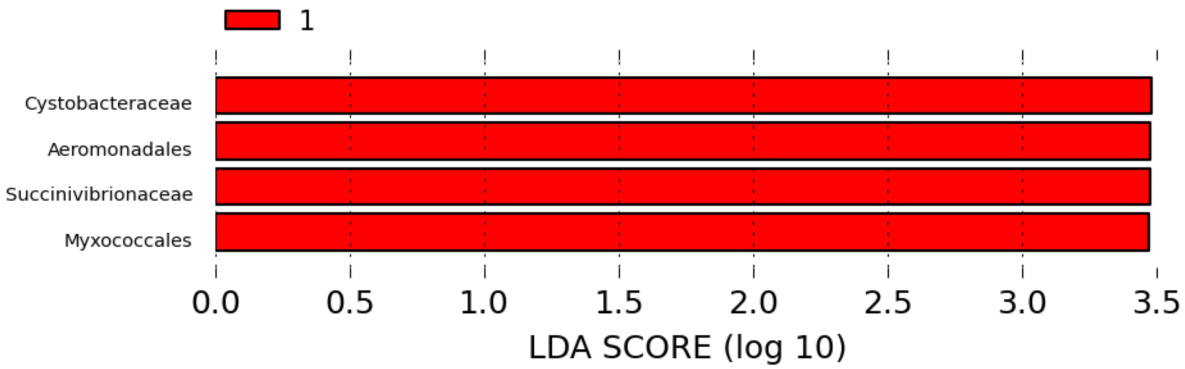

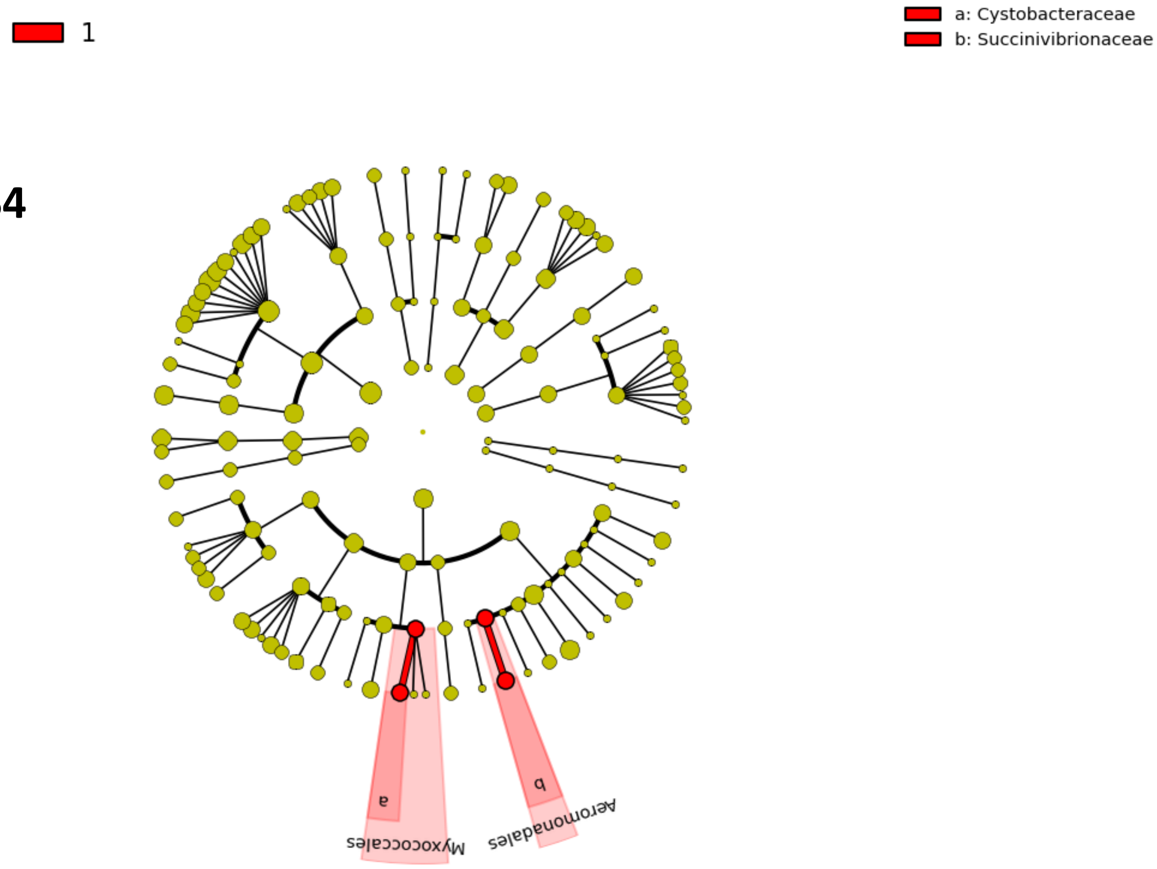

Figure S4

Bacterial Families in mucosa from patients hospitalized within 90 days

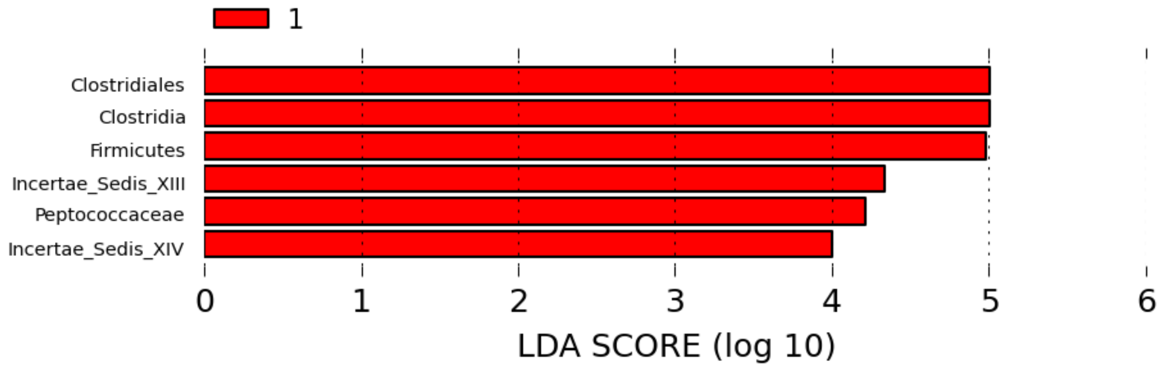

Figure S5

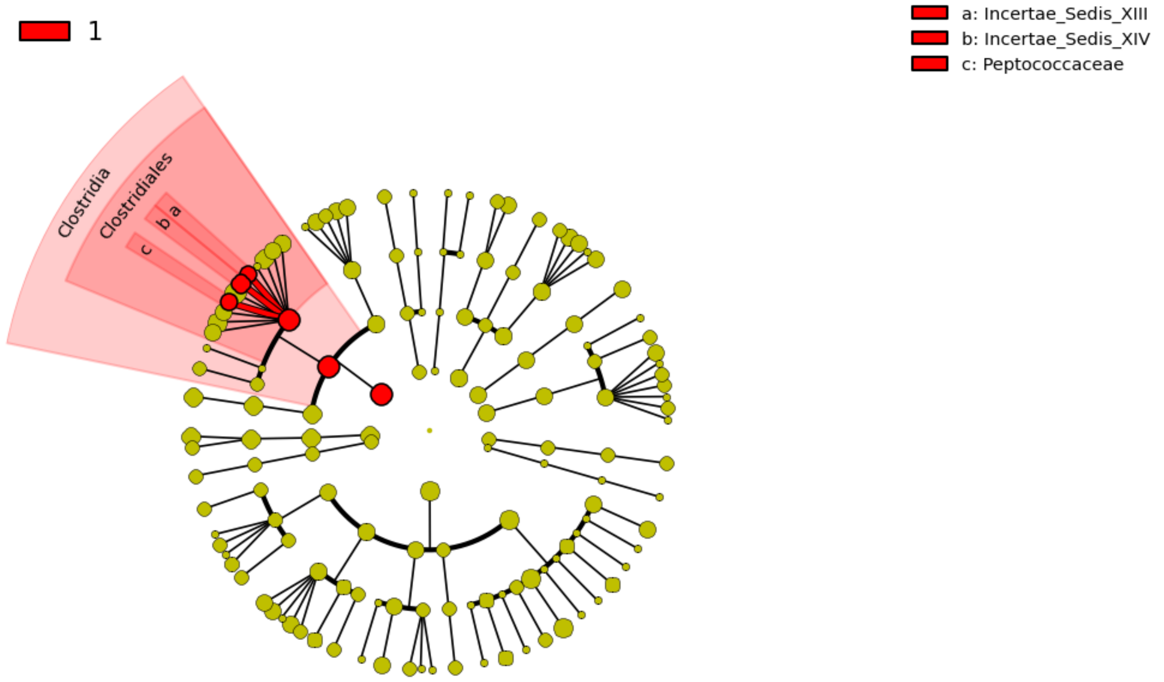

Bacterial Families in mucosa from patients with diabetes

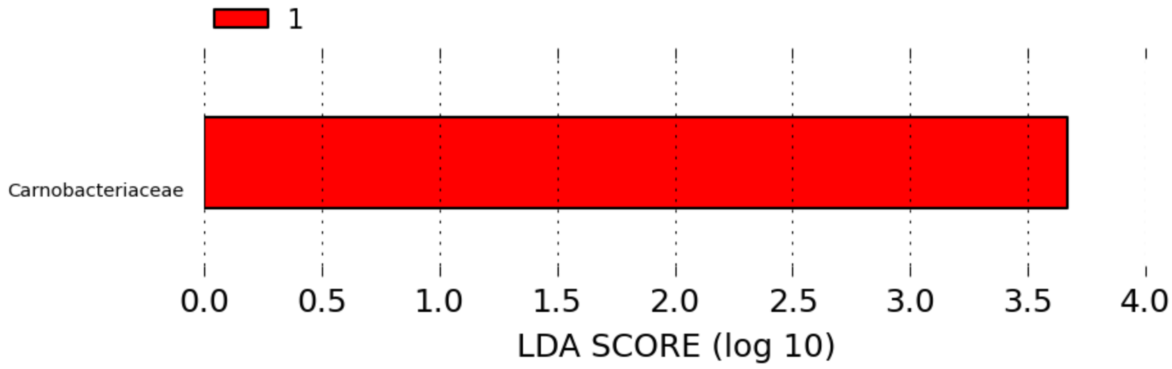

Figure S6

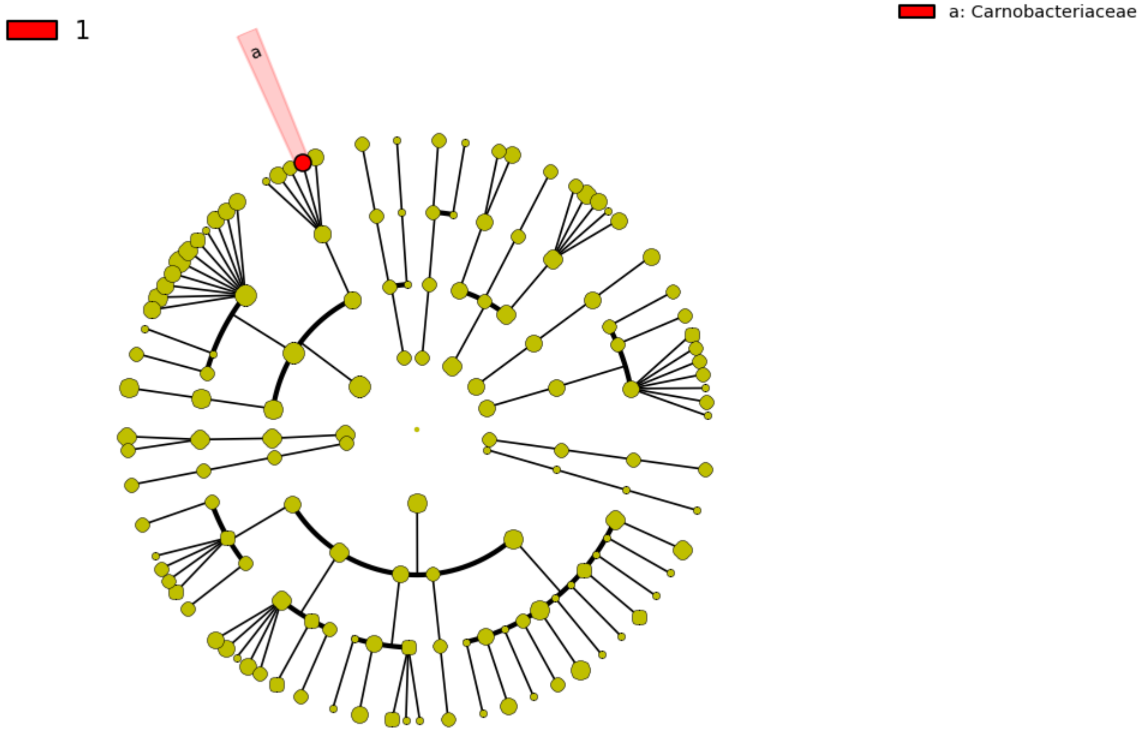

Bacterial Families in mucosa from patients treated with PPI
